# Evidence on Neurotoxicity after Intrauterine and Childhood Exposure to Organomercurials

**DOI:** 10.3390/ijerph20021070

**Published:** 2023-01-07

**Authors:** Lara Ferreira Azevedo, Nina Karpova, Bruno Alves Rocha, Fernando Barbosa Junior, Glenda Carolyn Gobe, Maria Fernanda Hornos Carneiro

**Affiliations:** 1 Department of Clinical Analyses, Toxicology and Food Sciences, School of Pharmaceutical Sciences of Ribeirão Preto, University of São Paulo, Ribeirão Preto 14040-903, SP, Brazil; 2 Kidney Disease Research Group, School of Medicine, Translational Research Institute, University of Queensland, 37 Kent Street, Woolloongabba, QLD 4102, Australia; 3 Department of Pharmacy, Faculty of Chemistry and Pharmacy, Pontificia Universidad Católica de Chile, Santiago 7820436, Chile

**Keywords:** methylmercury, ethylmercury, thimerosal, neurotoxicity, intrauterine exposure, childhood

## Abstract

Although the molecular mechanisms underlying methylmercury toxicity are not entirely understood, the observed neurotoxicity in early-life is attributed to the covalent binding of methylmercury to sulfhydryl (thiol) groups of proteins and other molecules being able to affect protein post-translational modifications from numerous molecular pathways, such as glutamate signaling, heat-shock chaperones and the antioxidant glutaredoxin/glutathione system. However, for other organomercurials such as ethylmercury or thimerosal, there is not much information available. Therefore, this review critically discusses current knowledge about organomercurials neurotoxicity—both methylmercury and ethylmercury—following intrauterine and childhood exposure, as well as the prospects and future needs for research in this area. Contrasting with the amount of epidemiological evidence available for methylmercury, there are only a few in vivo studies reporting neurotoxic outcomes and mechanisms of toxicity for ethylmercury or thimerosal. There is also a lack of studies on mechanistic approaches to better investigate the pathways involved in the potential neurotoxicity caused by both organomercurials. More impactful follow-up studies, especially following intrauterine and childhood exposure to ethylmercury, are necessary. Childhood vaccination is critically important for controlling infectious diseases; however, the safety of mercury-containing thimerosal and, notably, its effectiveness as preservative in vaccines are still under debate regarding its potential dose-response effects to the central nervous system.

## 1. Introduction

Mercury (Hg) is a natural hazardous pollutant occurring in different chemical forms: elemental Hg (Hg^0^); inorganic Hg compounds (Hg^+^/Hg^2+^) and organic Hg compounds, as methyl and ethylmercury (MeHg and EtHg, respectively) [1]. Many aspects associated with Hg exposure, such as individual characteristics (e.g., age or developmental stage), the Hg chemical form, as well as dose, route and duration of exposure, can produce particular degrees of toxicity [2]. It is known that human exposure to organic forms of Hg (such as MeHg and EtHg) occurs mainly through contaminated fish intake and administration of Hg-containing vaccines [3,4]. For infants, Hg oral exposures depends on the mother’s lifestyle and food consumption, since Hg intake occurs mainly through breastfeeding, while non-oral exposure heavily relies on vaccination schemes in developing countries, where thimerosal-containing vaccines (TCVs) are in routine use [5]. Both of these organomercurial compounds have been reported to exert cerebral toxicity jeopardizing the normal development and function of brain [6,7,8,9]. This study aims to critically review and discuss aspects regarding organomercurial kinetics as well as how MeHg and EtHg can impact the neurological system following both intrauterine and childhood exposure. In addition, prospects and future needs for research in this area are provided.

## 2. Exposure to Organic Mercury

Animal exposure to MeHg, including humans, occurs primarily through consumption of higher trophic level fishes and other marine mammals due to bioaccumulation and biomagnification processes along the food chain [10,11]. On the other hand, humans can also be exposed to organic Hg through the use of EtHg-containing vaccines [12]. Thimerosal—or sodium EtHg thiosalicylate—is made of 49.55% Hg by weight and has been added to multidose flask vaccines from 0.003% to 0.01% as a preservative [13,14,15]. Thimerosal was first synthesized in 1927 and has been used as a preservative in more than a few other pharmaceutical products such as cosmetics, eye drops, contact lens solutions, topical medicines as well as tattoo inks [15,16]. Although thimerosal is no longer used in the United Kingdom (UK) vaccines, and no TCVs were developed and approved in the US, this alkyl Hg compound is still added to flu vaccines administered to pregnant women, the elderly and infants in the US, and exposure to thimerosal via vaccination schemes is still prevalent worldwide, especially in developing countries, where it is used in many of the childhood vaccines such as hepatitis B (HepB), Haemophilus influenzae type B (Hib), diphtheria–tetanus–pertussis (DTP) and various influenza vaccines [17,18,19]. Exposure to EtHg from thimerosal occurs acutely by injection and the availability of pure thimerosal into the blood post-injection allows it to rapidly cross the blood-brain barrier (BBB), leading to an increased risk of Hg toxicity. In developing countries neonates receive their first vaccine with TCVs within 24 h, what can highly increase the Hg concentration in the blood, far exceeding that of the breastfeeding [20]. The amount of Hg in TCVs nominally ranges from 12.5 μg Hg to 25 μg Hg per dose (some vaccines could also contain >25 μg Hg per dose) and it is estimated that infants from developing countries receive about 200 μg of Hg from TCVs during their first six months of life [17]. Additionally, newborns can also be exposed to low concentrations of Hg, either as MeHg or EtHg, even before birth, since Hg can cross the placenta and can be readily bioavailable to be distributed to the newborn forming organs, including the developing brain. Gu et al. detected that the average Hg level in cord blood from 2316 neonates from Wujiang, China was 2.02 μg/L [21], a concentration that could be used as reference value of fetal Hg exposure from maternal placenta. Fetuses are particularly at a higher risk compared to adults, on a dose/weigh basis, and are more susceptible to adverse effects from Hg, being that it is not possible to estimate a safe level of maternal Hg transfer via placenta [22]. Currently, there are no exposure limits available for Hg exposure through TCVs administered. Therefore, it is critical that regulatory bodies propose those limits of exposure, accounting for exposure in utero, newborns and during childhood, since the developmental period of exposure is important regarding the risks for neurotoxic effects, since organomercurial compounds can result in health adverse effects even at low exposures [23,24,25,26,27].

As MeHg exposure occurs mainly by dietary fish consumption, MeHg poisoning is most probable to occur in consumers of contaminated fish and marine mammals. Effects from oral poisoning may result from intake of a single high dose or repeated exposure to low doses. To date, one of the most catastrophic ecological disasters that culminated in Hg poisoning occurred in May of 1956 in Minamata Bay, Japan, where people consumed fish and shellfish contaminated with MeHg formed from mercury sulphate released by an acetaldehyde chemical producing plant [28]. The marine products in Minamata Bay contained high levels of Hg—between 5.61 to 35.7 mg/kg—and the total amount estimated of Hg daily intake by fish consumption was between 2 and 5 mg for several months following the tragedy [28,29]. Another possible MeHg mode of poisoning is the direct contamination of food when it is used as a pesticide in agriculture, another aspect of public health concern additional to environmental exposure. Another example that illustrates this type of poisoning is the Iraq Neuropathy, a large epidemic that appeared in rural areas in Iraq in 1972. MeHg poisoning resulted from use of wheat grain treated with MeHg-fungicide to make bread. The grains were intended for planting and not directly for human consumption [30]. Neurodevelopmental toxicity was also reported in infants and neonates from pregnant women who were exposed to MeHg in both Minamata and Iraq, evidencing the ease of MeHg placental transfer [30]. There have also been several acute EtHg poisoning episodes of children in countries such as Iraq, Romania, China and Ghana, mainly caused by ingestion of food contaminated with EtHg chloride, such as rice and maize (reviewed in [31]).

## 3. Organomercurials Kinetics

The different forms of Hg have specific kinetic outlines which are responsible for the disparity in systemic distribution, patterns of biological effect and toxic potency [32,33,34]. It is known that Hg is easily absorbed into the blood stream when a parenteral thimerosal-containing vaccine is administered [4,35]. This was also observed following oral exposure to thimerosal in *Wistar* rats [36]. Despite the recent efforts in studying EtHg kinetics, there is less information on the biodistribution of Hg after exposure to this Hg species in comparison to MeHg [11,34,37]. After oral exposure to MeHg, approximately 95% of the Hg is distributed to all tissues within 30 to 40 h [38,39]. Additionally, following distribution, almost 5% of the absorbed dose continues in the blood, more specifically within red blood cells but also within the plasma compartment (at approximately 5% of that found in red blood cells) in which Hg is regarded as more bioavailable for producing effects [38,40]. It is believed that inorganic Hg (InoHg) is the predominant chemical form of Hg in the plasma of populations exposed to low levels of Hg, either to InoHg or MeHg, potentially from infrequent seafood consumption and mercury-containing amalgams [11,41,42,43]. In contrast, after evaluating Hg species in the plasma of individuals highly exposed to MeHg through fish consumption, Carneiro et al. reported that MeHg accounted in average for almost 40% of the total Hg in this blood compartment [44]. More specifically, when participants were analyzed independently, 22% of them presented a higher percentage of MeHg in plasma (≥50% of the total plasma Hg levels) [44]. In addition, plasma MeHg was correlated to a greater extent with toxic outcomes than plasma InoHg, suggesting that MeHg is the best and most reliable internal dose biomarker for Hg in chronically and highly Hg-exposed individuals. In agreement with these findings, several other investigators have demonstrated positive associations between detectable plasma Hg concentrations and adverse effects in MeHg-exposed subjects [45,46,47].

**Figure 1:**
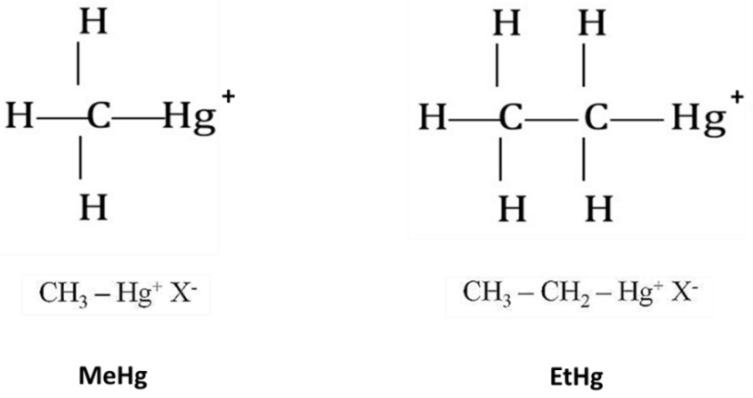
Chemical structures of methylmercury (MeHg) and ethylmercury (EtHg).

Due to the similarity in their chemical structure (Figure 1), kinetics and toxic effects of EtHg have been regarded for a long time to be similar to those of MeHg. However, differences in the biodistribution levels have been observed between these organic Hg species. For instance, monkeys and rats exposed to EtHg presented much higher levels of InoHg in the kidney and brain when compared to animals exposed to MeHg [36,48,49,50,51]. Still, more than 50% of Hg found in the blood, heart, brain, liver and kidney was in the inorganic form just after half an hour post thimerosal exposure [34]. These findings have been explained by the far more rapid biotransformation of EtHg into InoHg compared to that of MeHg [33,34,52]. In contrast, MeHg demethylation has been described to occur very slowly. Demethylated MeHg is the InoHg found in tissues probably as a result of reabsorption of part of the demethylated species [52].

MeHg is able to affect fetal brain development through transportation by the amino acid transporter subunits L-type amino acid transporter (LAT) 1 and rBAT (related to b^0,+^ type amino acid transporter). The efflux transporter multidrug resistance-associated protein (MRP) 1 is also involved in MeHg toxicokinetics [53]. Mercury conjugated to glutathione is able to be transported by the amino acid transporters located at the apical side of the syncytiotrophoblast while this conjugate is effluxed via MRP1 localized to the basal side of the syncytiotrophoblast. This explains why mercury is transported primarily towards the fetal side where it can exert toxicity, including neurotoxicity, during MeHg exposure [53]. MeHg accumulates more in the brain than in other tissues due to its lipophilic nature and chemical similarity with the amino acid L-methionine which is regularly interchanged by cells and blood [54,55]. In brain, there is evidence showing that the transportation occurs via the L-type large neutral amino acid transporter (LAT1) through a complex formation of MeHg with cysteine [56]. The same has been reported for EtHg, where C6 rat glioma cells were found to be significantly protected against the toxicities induced by both cysteine complexes of Hg (MeHg-S-Cys and EtHg-S-Cys) following the administration of L-methionine, in comparison with MeHg and EtHg alone [37]. Moreover, thiol-conjugates may also be involved in Hg transportation in tissues other than the brain, for example, the small intestine epithelium, liver and kidneys [55,57,58]. Although the exact mechanisms need further clarification, it is most probably that Hg removal from the brain occurs as a result of the co-transport of sulfur-containing compounds such as glutathione [59,60]. Information from experiments with mammals indicate that mercury from amalgam (elemental Hg) and MeHg can cross the placenta to be deposited in fetal tissues, primarily in fetal kidneys and liver [61,62,63,64]. Regarding thimerosal/EtHg no studies have been found. Therefore, information on the exact concentration of mercury or percentage of delivered dose and what is the main transferred Hg species that can actually cross the placenta and enter the newborn brain to cause the damage following exposure to EtHg or thimerosal is sparse.

**Figure 2:**
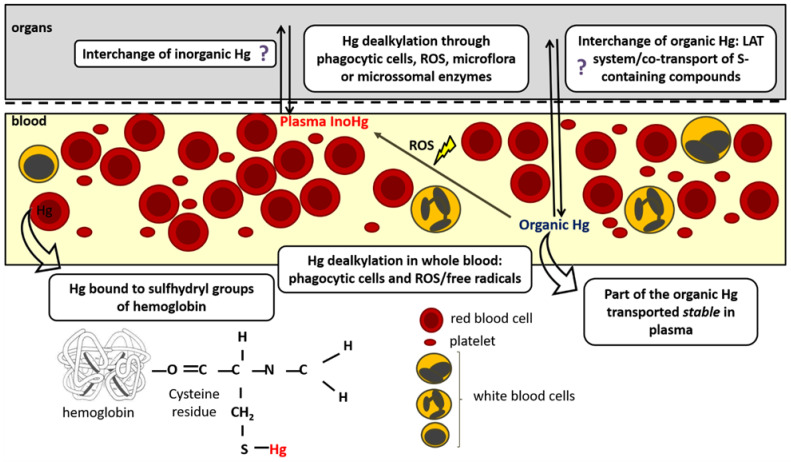
Main (and potential) processes involved in mercury dealkylation and transport between blood and organs. Organomercurial dealkylation is mediated in vivo by the intestinal microflora, microsomal enzymes as well as phagocytic cells (polymorphonuclear leukocytes, monocytes and macrophages). Conversion of organic Hg into InoHg can also occur through ROS and free radical formation. This conversion is known to occur far more rapidly in blood than in plasma—a likely less oxidative environment where organomercurials can be transported unmodified. Once in whole blood, EtHg (and potentially MeHg) can either bind to Hb cysteine residues and be converted into InoHg, be translocated and kept held unchanged in tissues, or be returned to plasma/red blood cells (based on [34,74]) findings). Question mark indicates processes that need to be proven (potential).

In vivo organomercurial dealkylation is considered a detoxification process [49,65,66] and it is probably mediated by the intestinal microflora [67,68], phagocytic cells such as polymorphonuclear leukocytes, monocytes and macrophages as well as microsomal enzymes [69,70,71]. There is also evidence that the conversion of organic Hg also occurs through reactive oxygen species (ROS) formation [34,69,72]. Specifically, Suda et al. have shown that neither the superoxide anion nor hydrogen peroxide alone can break the Hg-carbon bridge in MeHg [70]. In comparison, the hydroxyl radical (OH•) is able to promote dealkylation, mainly due to its high reactivity and low selectivity [73]. Furthermore, the EtHg molecule has a higher susceptibility to OH• attack, which could explain its greater and faster conversion into InoHg in comparison to MeHg [34,69]. EtHg biotransformation is much more significant in whole blood/erythrocytes compared to its minimal conversion in plasma, probably due to the presence of cells, hemoglobin (Hb) and a greater number of proteins and other compounds, while plasma is a less oxidative environment. In fact, in vitro experiments have demonstrated the potential interaction between EtHg^+^ (from thimerosal) and Hb cysteine residues (Hb-cys93) in a 2:1 stoichiometry ratio (thimerosal:Hb), resulting in Hb structure modification which in turn decreased its oxygen binding capacity [74]. A recent study confirmed that thimerosal concentrations as low as 1.25 µM can reduce the oxygen uptake by Hb through interactions with specific free cysteine residues in Hb, leading to conformational changes in the protein structure [75]. These studies highlight the high toxicity potential of EtHg to biological systems, due to its high binding capacity to Hb. In summary, once in blood, EtHg can be either captured by red blood cells and converted into InoHg, be held unchanged in protein-rich tissues, or be returned to plasma/red blood cells [34]. Figure 2 depicts the known and potential processes involved in the organic Hg dealkylation and transport between blood and organs.

Some authors state the dealkylation of MeHg into InoHg occurs in the brain with the ratio of InoHg to MeHg increasing with age [76,77]. Potential explanations have linked the MeHg demethylation processes with a possibly limited ability of InoHg to be eliminated across the blood brain barrier (BBB) due in part to an insoluble complex formed between InoHg and Se [39,78,79]. However, a study using labeled Hg isotopes found no measurable conversion of MeHg into InoHg in mink brains, unlike in the liver and kidney, suggesting that brain demethylation is insignificant [80]. Also, following EtHg exposure, two main scenarios may explain the presence of InoHg in the brain: (i) EtHg crosses the BBB and is converted to InoHg within this tissue; or (ii) InoHg is sequestered from blood to brain, since studies have reported InoHg in this tissue after exposure to InoHg [34,81,82,83,84].

After exposure to MeHg, the formed InoHg is eliminated through urine and feces, whereas MeHg itself is first converted to InoHg, conjugated to bile acids (which can be reabsorbed) and excreted through feces [11]. After performing a follow-up study of 98 days, Thomas et al. suggested that feces is the main route of excretion of Hg following acute exposure to MeHg in *Long Evans* rats, accounting for approximately 50% of the dose, with about 20% corresponding to InoHg [85]. Also, the organic:inorganic Hg ratio in the feces of guinea pigs was found to be 1:3 after subacute exposure to MeHg [86]. Excretion of Hg via the urinary tract was considerably lower (3 to 7% of the dose) [85]. In comparison, in non-exposed subjects, the amount of Hg excreted in feces is slightly higher than that found in urine, with a very similar MeHg concentration found in both feces and urine [87]. For EtHg, one can indirectly assume that a significant part of the Hg excreted after thimerosal exposure is potentially eliminated by urine, since most of the Hg determined experimentally in the kidney after acute thimerosal exposure was in the inorganic form [34]. Pichichero et al. described substantial excretion of Hg via feces rather than urine in infants receiving vaccines [88]. However, in this study, urine was collected as spot samples (at one time point interval after vaccination) and urinary Hg levels were reported in concentration and not in absolute amount of Hg excreted (i.e., without volume correction). This discrepancy in data interpretation may arise due to different follow-up evaluations (exposure and duration, frequency of administrations, route and dose of exposure) and evidences the need for evaluation of chronically and highly exposed individuals in order to clarify organomercurial elimination kinetics.

The half-life of Hg in blood after EtHg exposure has been demonstrated to last between 5.6 to 8.8 days in mice [34,49,88,89,90], very close to those reported for human adults (5.6 days) and newborns (6.3 and 7 days) following thimerosal administration [88,89,90]. Therefore, although the biological interaction of Hg may differ between humans and other animals [79], published literature on Hg half-life in blood following exposure to thimerosal strongly indicate similarities between the two categories. Carneiro et al. also reported Hg half-lives of 10.7 for brain, 7.8 for heart, 7.7 for liver and 45.2 for kidney [34]. In contrast, reported Hg half-lives in human blood ranged from 35 to 100 days after MeHg exposure [91,92]. For the majority of the organs, reported Hg half-lives are also longer considering MeHg exposure in comparison to EtHg exposure, with the exception of the kidney, which is influenced by the formation of InoHg following EtHg exposure, whose Hg half-life is estimated at approximately 58 days [11,34,93,94].

## 4. Neurotoxicity Effects following Intrauterine and Childhood Exposure to Methylmercury

Most of the documented MeHg neurotoxic effects are associated with developmental exposure to this compound. The major risk for human neurodevelopment was found after in utero MeHg exposure (reviewed in [95]). During embryogenesis, the central nervous system rapidly develops and is highly vulnerable to subtle environmental changes and toxic effects. MeHg passes through the physiological barriers, such as the placenta and the BBB [96]. The higher Hg accumulation in the brain during embryonic exposure, compared to that observed at later stages of development, might reflect facilitated MeHg transfer across the poorly developed BBB [97]. Moreover, the maturation of the BBB itself may be impaired after MeHg exposure, probably due to astrocytic damage [98]. These findings could explain the high sensitivity of the developing brain to MeHg.

**Figure 3:**
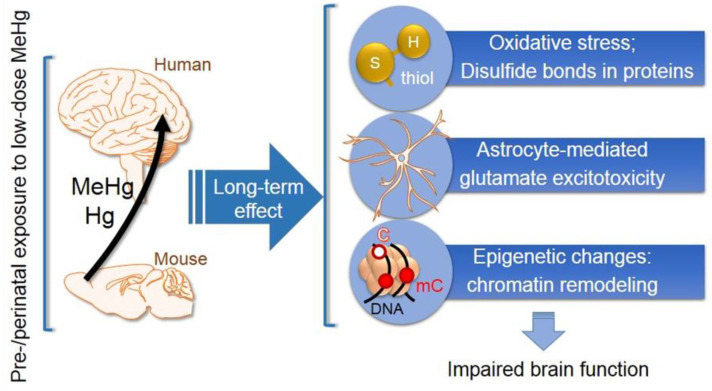
The main mechanisms for the long-term effect of pre/perinatal exposure to methyl mercury (MeHg) on impaired brain function. Chronic exposure to MeHg leads to brain-specific accumulation of MeHg and inoHg in the developing fetus. MeHg may exert its effects on cell function and gene expression by: (1) binding to the thiol groups in glutathione, seleno-dependent enzymes and other proteins pertaining to the antioxidant system; (2) inducing astrocyte-dependent glutamate excitotoxicity and (3) by epigenetic reprogramming of gene expression through chromatin remodeling. A nucleosome composed of DNA and histone octamer is shown; epigenetic changes in DNA methylation are schematically represented by C (unmethylated cytosine) and mC (methylated cytosine).

The key role of MeHg in neurotoxicity is attributed to the covalent binding of MeHg to sulfhydryl (thiol) groups of proteins and other molecules, such as GSH [99]. As mentioned above, the binding to cysteine allows MeHg to be transferred through the BBB because the complex MeHg/L-cysteine is structurally close to L-methionine, a substrate for LAT1 enzymes [100]. Additionally, thiol groups are also critical for protein post-translational modifications. Therefore, when MeHg binds to thiol groups of proteins it exerts toxic effects by affecting numerous molecular pathways, such as glutamate signaling (the main excitatory neurotransmitter), heat-shock chaperones (e.g., Hsp90), in addition to the antioxidant glutaredoxin/glutathione system, which includes glutathione (GSH) and seleno-dependent enzymes, such as glutathione peroxidase (GPx) (Figure 3) [101,102]. The in vitro MeHg-induced inhibition of glutamate uptake, specifically by the astrocyte glial cells, resulted in increased extracellular glutamate levels that could activate N-methyl D-aspartate (NMDA) receptors and rise intracellular Ca^2+^ concentrations, potentially leading to neuronal cytotoxic injury (Figure 3) (reviewed in [103]). However, to draw this conclusion, neuronal cell cultures should have been used for the comparison with astrocytic cell cultures.

In vivo studies on adult monkeys also showed that chronic exposure (6 or 12 months) to low-dose MeHg (50 µg Hg/kg/day) decreased the number of astrocytes in the thalamus without having a significant effect on other cell types [104]. Together, it was suggested that astrocytes are especially sensitive to MeHg toxicity, whereas the neuronal damage is secondary to astrocytic dysfunction [102]. In contrast, an in vitro study suggested that neurons are more susceptible to Hg species-induced cytotoxicity than human astrocytes [6]. These researchers found dehydrogenase activity, lysosomal and membrane integrity being affected by much lower concentrations of the different Hg species in study (HgCl_2_, EtHg and MeHg) in neurons than in astrocytes. Although these studies implicate neurons and astrocytes in MeHg-induced toxicity, there is an obvious knowledge gap in the cause–effect relationships between these cell types that requires further research.

Oxidative damage to the cells is among the primary causes of neurotoxicity, including MeHg-induced neurotoxicity (Figure 3) [101]. MeHg exposure may result in decreased GSH levels in cultured neurons and astrocytes, therefore reducing ROS scavenging capacity [105], while it may also increase ROS production by mitochondria [106]. Several studies associate chronic oxidative stress and ROS accumulation with cognitive impairment, anxiety and depression-like behaviors in humans and rodents [107,108,109]. Furthermore, growing evidence from animal studies suggests that transient pre/perinatal exposure to low levels of MeHg (similar to the exposure levels in high fish-consuming human populations) leads to chronic oxidative stress induction associated with decreased expression of key antioxidant enzymes, such as glyoxalases, glutathione peroxidase and/or reductase, in the brain of weanling [101] and adult [110] mice. In healthy developing mouse brains, the levels of GSH and glutathione peroxidase gradually increase during prenatal development; this process may be required for neuroprotection against increased oxygen metabolism and ROS production that occur after birth [111]. Thus, the MeHg-induced impairments of the developing antioxidant system can render the brain more sensitive to damaging effects of postnatally produced ROS [101]. In accordance with this statement, a study in mice showed that perinatal exposure to low-level MeHg strongly delayed the development of parvalbumin (PV) interneurons across the brain at the pre-weanling age P17, and promoted the rapid, dramatic increase in the formation of perineuronal nets (PNNs) around the neurons, including PV interneurons, from P17 to post-weanling age P24 [112]. PV is a calcium-binding protein expressed in GABAergic fast-spiking interneurons [113], the number of which greatly increases during the early stages of development of newborns [112]. PV interneurons are critical for the formation of inhibitory brain circuits, the development of experience-dependent neuronal plasticity, learning and memory [114]. Cabungcal and co-authors reported that oxidative stress is especially damaging for the development of PV-interneuronal subtypes [115,116]. PNNs are composed of extracellular matrix proteoglycans and linking proteins, particularly expressed around GABAergic interneurons [117]. They control PV interneurons maturation and inhibitory brain circuits [118] and protect neurons from oxidative damage [115,116,119]. Umemori and co-authors suggested that the rapid production of PNNs from P17 to P24 might be a protective mechanism of the brain against MeHg-induced oxidative stress [112].

Although the postnatal brain might develop some protective mechanisms against prenatal MeHg-induced toxicity, there is growing evidence from animal and human studies about irreversible long-lasting damage of MeHg to the nervous system. Ceccatelli and co-authors provide an excellent overview of these studies [120]. The developmental MeHg exposure promotes long-term deficits in brain functioning that become evident with age (“silent neurotoxicity” [121]). These deficits include cognitive performance, attention and memory, and neuropsychiatric disorders [122,123,124]. Decreased adult hippocampal neurogenesis, which is critical for adult neuronal plasticity [125], could be one of the mechanisms for the development and long-lasting maintenance of these deficits [120]. MeHg-induced impaired mitochondrial function and neural stem cells oxidative damage has been reported [26]. Moreover, perinatal MeHg exposure in mice induced epigenetic reprogramming of gene expression in the hippocampus [126,127]. Pathological epigenetic reprogramming early in life may impair neuronal plasticity and trigger the development of brain disorders in adult humans and rodents (Figure 3) (for one of the latest reviews, see [128]). Epigenetic mechanisms such as chromatin remodeling, DNA methylation and histone modifications, non-coding RNAs and transposable elements, control short- and long-term alterations in gene expression without changes in DNA sequence (reviewed in [129]). Epigenetic mechanisms of genome plasticity are vitally important for brain functioning because epigenetic changes are able to influence gene expression in non-dividing cells, such as neurons [127,128,129].

The implication of genetic factors in MeHg-induced neurotoxicity might probably resolve at least some discrepancies between numerous epidemiological studies of human early-life MeHg exposure in fish-consuming populations around the world, including the Faroe Islands [130], Brazil [131,132], Seychelles [133], Quebec [134], Madeira and Japan [135]. Interestingly, the genetic polymorphism in apolipoprotein E (APOE), a major protein transporter in the brain, was significantly correlated with Hg cord blood concentrations and neurodevelopmental impairment in children in Taiwan, where MeHg from fish was assumed as the main source of Hg [136]. Moreover, in a low-fish consuming population from the UK, a possible genetic predisposition to MeHg-induced cognitive deficits was associated with single-nucleotide polymorphisms (SNPs) within genes for paraoxonase 1, progesterone receptor, as well as transferrin and brain-derived neurotrophic factor (Bdnf) [137]. In a more recent study, Morris and colleagues reviewed studies which demonstrate that epigenetics and genetic factors, such as polymorphisms to single genes (e.g., metallothionein, coprophorphyrogenoxidase, ATP-binding cassette transporter, BDNF and APOE) as well as to GSH antioxidant system-related genes, increase the susceptibility of children to the neurotoxic effects of InoHg and MeHg [138]. In this study, the authors also concluded that Hg exposure may be associated with the development of autism spectrum disorders (ASD) in children, particularly on those in which some type of mutation in GSH system-related genes have been detected, since several reviewed studies evidence positive associations between GSH system abnormalities and ASD onset [138]. In animal mouse models, the deficiency in Reelin, an extracellular matrix protein critical for brain development, was identified as a susceptibility risk factor for prenatal MeHg-induced toxicity [139], whereas overexpression of TrkB (a Bdnf receptor) could protect from MeHg-dependent depression-like behavior but not anxiety [110]. Because large epidemiological studies that include genotyping are highly costly, the preclinical studies in experimental animals currently provide an invaluable tool for elucidating molecular mechanisms of MeHg- as well as EtHg-induced neurotoxicity.

## 5. Neurotoxicity Effects following Intrauterine and Childhood Exposure to Ethylmercury

As discussed above, currently the main source of EtHg exposure is as thimerosal preservative in vaccines, since the EtHg-containing fungicides should have been eliminated from use [140]. It is noteworthy that the administration of TCVs, which results in acute EtHg exposure in pregnant women, newborns and infants, is still a major concern faced by less developed countries, where there are not public policies to support the use of thimerosal-free vaccines, while in more developed countries this ingredient was significantly withdrawal from infant vaccine formulations to avoid any possible associated exposure risks [20,141]. Therefore, more robust evidence from populational studies addressing the neurotoxic effects associated with acute exposure to TCVs-EtHg are of utmost importance to support vaccine-policymakers’ decisions in developing countries.

The majority of studies, including large epidemiological studies, try to explore the causal relationship between early-life exposure to Hg from TCVs and ASD (for detailed review, see [142]). This relationship was first reported in 1998 in a Lancet article which was later retracted and, therefore, not cited here. Most studies report absence of correlation between human thimerosal exposure and childhood neuropsychological outcomes including ASD in the UK [143], Denmark [144,145] and in a review paper [146]. A few negative associations of thimerosal content in vaccines include lower finger-tapping scores in 10–11-year-old girls in Italy [147], speech disabilities in boys and girls, and lower verbal IQ in girls [148], and tics in boys [149] in 7 to 10 years old children in the USA. The authors also reported several beneficial outcomes associated with vaccinations, such as better fine motor coordination [148]. However, not all studies were “case-control”, i.e., included the control group of children that received the thimerosal-free vaccines, which is a recurrent limitation in epidemiological studies which investigate thimerosal exposure in children. Nonetheless, the absence of negative effects of TCVs on neuronal pathology and ASD-related behavior has been supported using a monkey model [150,151]; however, the authors did not access the Hg levels in vaccinated animals. Several case-control studies in USA children associated a risk of delays in development or ASD with thimerosal; the cases were individuals with pervasive developmental disorders, delays (reading difficulties, dyslexia, language disorders, etc.) or ASD, whereas the controls were healthy children. However, most of these studies were led by Dr. Mark Geier, whose research has been questioned [152]. Apart from evidence presented by Geier´s group, relevant literature on case-control studies does not support a causal association between mercury exposure from the preservative thimerosal and increased risk of ASD [153,154,155]. Also, according to a comparative pharmacokinetic estimate of mercury in U.S. infants following yearly exposures to inactivated influenza vaccines containing thimerosal, TCVs can be considered safe [156]. In the same way, the meta-analysis performed by Yoshimasu et al. reported no material associations between thimerosal exposure and increased risk of ASD or ADHD [157]. This is potentially related to the low-level exposure to Hg that occurs through TCVs and the rapid elimination of EtHg from blood.

According to Modabbernia et al., future risk assessment studies of ASD would benefit from a developmental psychopathology approach, prospective design, precise exposure measurement and should take into account the dynamic interplay between gene and environment by using genetically informed designs [158], since a number of epidemiological studies on this issue is limited to observations made at an early stage. Therefore, focusing on reliable timing of exposure in relation to critical developmental periods as well as to genetic vulnerabilities of developmental disorders is of great importance for better understanding the susceptibility to EtHg exposures [157].

In order to solve the discrepancies mentioned above, the analysis of potential genetic or environmental risk factors of susceptibility to EtHg and putative molecular mechanisms of possible EtHg-induced neurotoxicity, experimental non-primate models (e.g., rodent) could be helpful. Some of the already-documented neurotoxic effects after in utero/perinatal exposure to thimerosal through in vivo experimental studies are shown in Table 1. Different neurotoxic outcomes have been described to arise following exposure to thimerosal such as neural development delay, social interaction deficiency (autistic-like behavior), delayed auditory response, increased levels of oxidative stress biomarkers, hormonal deregulation, altered susceptibility to neurotransmitters, etc. In comparison to epidemiologic data—where some of the studies evidence the absence of association between EtHg exposure and neurotoxic effects—there is a myriad of experimental studies that report such effects. This fact might arise from the use of high doses that do not represent the real scenario, or more remotely due to the current devaluation of studies with negative results. For instance, among the studies investigating neurotoxicity following the exposure to thimerosal in low doses [24,159,160] only the study of Olczak et al. showed toxicity (locomotor activity impaired in males receiving 12 µg Hg/kg at postnatal days 7, 9, 11 and 15) [160]. The other studies using low doses showed no effect at the lowest dose used [24,159]. When comparing how the exposure to thimerosal/EtHg is carried out experimentally in non-human puppies versus the vaccination schedule of human babies, one can assume the in vivo studies are relevant since they expose rats on postnatal days 1 to 15. This is relevant because the majority of vaccines are administered during the first twelve months of age, what would correspond to almost 14 days in rats [161]. Nevertheless, studies evaluating neurotoxicity induced by EtHg/thimerosal following exposure in utero are much rarer in comparison to postnatal exposure.

To date, only a few rodent studies examined a potential neurotoxic role of EtHg and there are rare studies aiming to mechanistically investigate the pathways involved in the neurotoxicity of EtHg/thimerosal. One of the first studies that highlighted the importance of genetic profile for EtHg sensitivity showed that the autoimmune disease-sensitive mouse strain *SJL/J* exhibited profound neurodevelopmental deficits (growth retardation, locomotor activity, hippocampal distortion and enlargement) after thimerosal exposure, compared with strains without autoimmune sensitivity (*BALB/cJ*, *H-2d*, or *C57BL/6J*, *H-2b* mice) [159]. However, another study using the same mouse strain did not confirm these findings [24]. Later, the neurotoxic effects of thimerosal, at doses similar to those used in vaccines, reported decreased pain sensitivity (nociception) [162] and significant neuroanatomical alterations in the hippocampus, cerebellum, temporal cortex in six-week-old young adult rats [163]. This same research group documented thimerosal dose-dependent and sex-dependent impairment of locomotor activity, anxiety, and social interactions, which were associated with reduced striatal dopamine D2 receptors, probably by inactivating its functional thiol groups [160]. Moreover, the exposure of neonatal mice to thimerosal doses 20 times higher than that used in humans resulted in marked neurodevelopmental delay and deficiency in social interaction in adulthood, suggesting an ASD-like phenotype [164]. Moreover, the high-throughput RNA sequencing suggested the involvement of gonadotropin hormone signaling (up-regulated), immune genes (down-regulated) and axonal guidance signaling (differentially affected) in higher-dose thimerosal-induced ASD-like behavior [164]. However, this study was developed using concentrations of Hg as thimerosal 20 times higher than that used in regular Chinese infant immunization during the first 4 months of life. Finally, one of the latest experimental studies documented to date trying to elucidate possible mechanisms associated to EtHg-induced neurotoxicity showed that this compound can cause a concentration-dependent (3 to 30 µM) ependymal cell cilia movement inhibition in *ICR* mice brain slices, with an IC50 of 5.80 µM for inhibition curves, which is around concentrations previously described for human poisoning [165]. Although this study did not investigate the effects of EtHg following pre- or perinatal exposure, the authors concluded that the inhibition of ependymal cell cilia movement might be particularly damaging during prenatal exposure, since during this exposure period the underdeveloped BBB is more susceptible to EtHg and the cerebrospinal fluid flow—which is solely dependent on cilia motility—is highly important for active migration and proliferation of neurons [165].

**Table 1:** Neurotoxic effects after in utero/perinatal exposure to thimerosal/EtHg through in vivo experimental studies.

Biological System	Route	Exposure Design and Dose	Outcomes	Reference
*FVBN_NJ* mice	SC ^1^	Pups were injected on postnatal day 1 (P1), P3, P5, and P9 with a 20-fold higher Hg dose than those used in current Chinese infant immunization schedule during the first 4 months of life (304, 238, 196, and 176 μg of Hg/kg, given respectively to each of the postnatal days)	The treated pups showed life neural development delay (eye-opening ratio), social interaction deficiency (autistic-like behavior), and inclination for depression. Neuropathological changes were also observed in the brain tissue of adult mice neonatally treated with thimerosal. High-throughput RNA sequencing of autistic-behaved mice brains revealed the alternation of a number of canonical pathways involving neuronal development and neuronal synaptic function as well as several gonadotropin hormone transcripts were strikingly up-regulated in thimerosal-treated males.	[164]
*Spontaneously Hypertensive Rats* (SHR) or *Sprague–Dawley* (SD) rats	SC ^1^	Dams were exposed to 200 μg thimerosal per kg during pregnancy (gestational days 10 to 15) and lactation (post-natal days 5 to 10).	Delayed auditory (startle response) was verified in SD neonates; decreased motor learning was registered for males (both SHR and SD) and also for SD females. Also, a significant increase in cerebellar levels of the oxidative stress marker 3-nitrotyrosine was found in higher levels in SHR females as well as in SD males than in controls. The activity of cerebellar type 2 deiodinase (converts thyroxine to the active hormone, 3′,3,5-triiodothyronine (T3)) was significantly decreased in SHR males exposed to thimerosal with Odf4—a gene regulated by the levels of T3—found to be overexpressed in comparison to controls.	[18]
*SJL/J* mice	combination of SC ^1^ and IM ^2^	Pups were injected on post-natal days 7, 9, 11, and 15 with: vehicle, 1 × thimerosal (cumulative dose = 39.8 μg Hg/kg representing the maximum Hg exposure, on a μg/kg basis, to which a child could have been exposed from vaccination if hepatitis B, diphtheria tetanus pertussis (DPT), and Hemophilus influenza B (HiB) were conserved with thimerosal) or 10 × thimerosal (10-fold higher cumulative dose: 390 μg/kg)	No significant behavioral alterations (i.e., social interaction, sensory gating, and anxiety) were produced by the treatment.	[24]
*SJL/J, C57BL/6J,* and *BALB/cJ* mice	IM ^2^	Pups were treated at postnatal day (P)7, P9, P11, and P15 with (1) thimerosal-only (14.2, 10.8, 9.2, or 5.6 µg/kg of ethylmercury per postnatal immunization day) or (2) thimerosal vaccines (thimerosal-preserved diphtheria, tetanus, acellular pertussis (DTaP, Lederle), and Haemophilus influenza B (HiB, Lederle) vaccines—EtHg load the same as group (1); or (3) Control (PBS)	Thimerosal-treated animals—either through thimerosal administration or through its content as vaccines—had no significant differences in any parameters measured and results were then combined for the analysis. *SJL/J* mice are known to be autoimmune disease-sensitive and showed growth delay, decreased locomotion, amplified response to novelty, and densely packed, hyperchromic hippocampal neurons with altered glutamate receptors and transporters. Strains resistant to autoimmunity—*C57BL/6J* and *BALB/cJ*—were not affected by thimerosal.	[159]
*Wistar* rats of both sexes	IM ^2^	Pups were injected on postnatal days 7, 9, 11 and 15 with one of the four different doses in study (12, 240, 1440, or 3000 µg Hg/kg) + vehicle	The locomotor activity was impaired in males at the lowest dose tested whereas in females this effect was only observed at the highest dose; animals of both sexes treated with the highest dose of thimerosal presented reduced rates of prosocial behavior and the frequency of asocial/antisocial interactions was increased and decreased, respectively, in males and females. For males, significant less striatal D2 receptors were found at the dose 12 µg Hg/kg while for females this was observed at 240 µg Hg/kg. No effects were documented considering spatial learning and memory.	[160]

^1^ SC: subcutaneous. ^2^ IM: intramuscular.

## 6. Conclusions

Taken all together, the current knowledge about EtHg-induced neurotoxicity is still controversial perhaps due to the lack of costly genetic assessment of the human populations studied, insufficient data about human subjects involved in the studies (such as the prenatal and early postnatal exposure to other environmental stressors, physical or chemical), and only a small number of animal studies that could provide molecular mechanisms of EtHg effects on brain development. Childhood vaccination is critically important for control of some infectious diseases; however, the safety of thimerosal and, notably, its effectiveness as a preservative at the used dose, is still under debate.

## Data Availability

No new data were created or analyzed in this study. Data sharing is not applicable to this article.
